# Inner Ear Organoids: A Hydrogel-Based Platform for Drug Screening and Deafness Modeling

**DOI:** 10.1007/s12264-025-01479-0

**Published:** 2025-10-21

**Authors:** Yuyu Cao, Xiaotao Liu, Renjie Chai, Zuhong He

**Affiliations:** 1 Department of Otolaryngology Head and Neck Surgery, Zhongda Hospital, School of Medicine, Advanced Institute for Life and Health, Southeast University, Nanjing, 210096 China; 2 State Key Laboratory of Digital Medical Engineering, Department of Otolaryngology Head and Neck Surgery, Zhongda Hospital, School of Life Sciences and Technology, Advanced Institute for Life and Health, Jiangsu Province High-Tech Key Laboratory for Bio-Medical Research, Southeast University, Nanjing, 210096 China; 3 Department of Otorhinolaryngology-Head and Neck Surgery, Zhongnan Hospital of Wuhan University, Wuhan, 430071 China

**Keywords:** Inner ear organoids, Hydrogel, Drug screening, Hearing loss, Disease modeling

## Abstract

This review highlights advances in inner ear organoids (IEOs) as a novel platform for drug screening and disease modeling, particularly for hearing loss. IEOs, derived from embryonic stem cells, induced pluripotent stem cells, or tissue-specific progenitors, provide a physiologically relevant alternative to traditional animal models. Significant progress has been made in utilizing various cell sources, extracellular matrix materials such as Matrigel and hydrogels, and methods for controlling microenvironments through biochemical and biophysical signals. Applications of IEOs in drug screening, disease modeling, and personalized medicine enable exploration of hearing loss mechanisms and therapeutic testing. However, challenges remain, including the incomplete maturation of cochlear cells and difficulty replicating *in vivo* environments. Future research should focus on optimizing IEO generation, incorporating microfluidic technologies, and advancing high-throughput screening to enhance drug discovery and clinical translation.

## Introduction

In traditional drug development, animal models and cell culture systems have long played a crucial role [1, 2]. However, these methods inevitably have certain limitations, such as individual differences in animal models, ethical concerns, and the inability of conventional cell culture systems to accurately simulate complex biological systems [3, 4]. With the continuous advances in tissue engineering and regenerative medicine, the use of organoids as platforms for drug evaluation and testing has become a hotspot of current research. Organoids were initially used to mimic complex biological structures such as the intestine and brain, as their construction process involves the extraction of stem cells from patients or model animals, followed by three-dimensional (3D) culture and the induction of differentiation into specific tissue or organ-like structures *in vitro* [5–7]*.* This approach more realistically simulates the physiological environment *in vivo*, thereby providing a more accurate and reliable experimental platform for drug development [8].

As an emerging branch in the field of organoids, inner ear organoids (IEOs) have garnered widespread attention in auditory research due to their unique structure and function. The inner ear is the core of the auditory system and is crucial for sound perception, speech recognition, and the development and cognitive functions of the nervous system, largely due to the function of different cells in the sensory epithelium [9–11]. However, the pathophysiological mechanisms of inner ear developmental abnormalities and sensorineural hearing loss are complex, and research into these phenomena is extremely challenging due to the limited number of endogenous hair cells (HCs) and the anatomical complexity of the inner ear deep within the temporal bone [12, 13]. Particularly in adults, the rapid degeneration of HCs following isolation further restricts the ability to study HC function and the regulation of gene expression *in vivo* [14]*.* Therefore, research on IEOs offers a highly efficient and reliable platform for studying inner ear development, regeneration, and the pathophysiological mechanisms of sensorineural hearing loss, as well as for exploring potential therapeutic strategies. In recent years, many researchers have successfully induced the growth of IEOs from pluripotent (PSCs) and mono‐potent stem cells [15, 16], and these organoids not only exhibit cellular compositions and structures similar to those found *in vivo*, but also demonstrate partial functional characteristics, enabling researchers to study the complex biological processes and pathophysiological mechanisms of the inner ear *in vitro* and to develop potential therapeutic methods [17, 18].

**Fig. 1:**
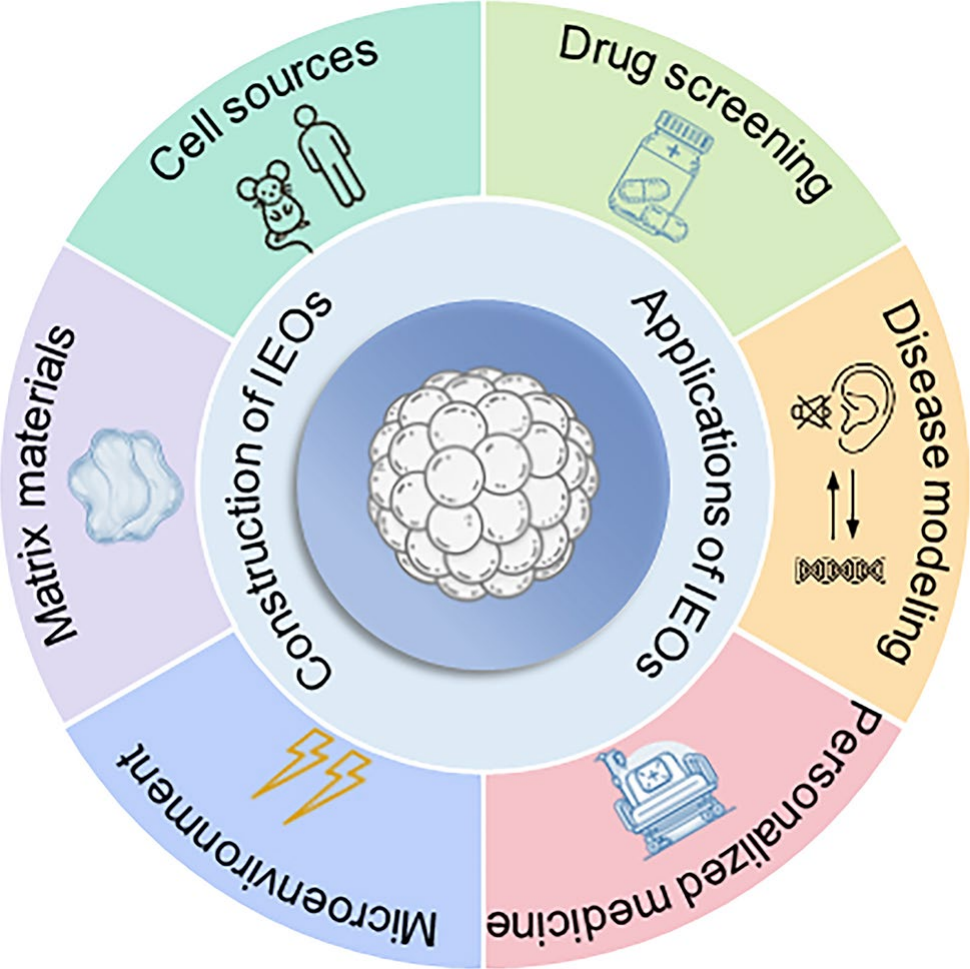
Schematic of the construction and applications of IEOs.

In this review, we present the latest research advances in the construction and application of IEOs (Fig. 1). We first discuss the different cell sources used to construct IEOs and the advantages and disadvantages of inducing their formation. Following this, we provide a detailed explanation of the role of traditional scaffold materials and discuss recent advances in novel materials for optimizing scaffold performance. After establishing the appropriate culture scaffolds, we further explore how to accurately mimic the inner ear microenvironment, including the control of biochemical and biophysical conditions. Finally, based on these discussions, we present the major applications of IEOs and assess the potential and future prospects of this technology.

## Construction of IEOs

### Cell Sources

#### Embryonic Stem Cells (ESCs) and Induced Pluripotent Stem Cells (iPSCs)

The cell sources for IEOs are diverse, mainly including ESCs, iPSCs, and tissue-specific progenitors (TSPs). Each source has its own unique advantages and challenges in organoid research and applications. Currently, studies using ESCs and iPSCs to generate IEOs are more widespread than those using TSPs. In 2010, Oshima *et al*. first demonstrated that functional inner ear HCs could be derived from ESCs and iPSCs [19], and Koehler *et al*. later introduced a method for inducing human PSCs into IEOs [16]. Their approach induced the formation of non-neural ectoderm using Matrigel, fibroblast growth factor-2, and SB-431542, followed by co-treatment with fibroblast growth factor-2 and LDN-193189 to induce the development of the otic-epibranchial progenitor domain. This method demonstrated that HCs derived from human PSCs exhibit electrophysiological properties similar to natural sensory HCs. Jeong *et al*. optimized these methods by adding growth factors at specific stages to induce IEO formation in a stepwise manner [20, 21]. This approach resulted in 3D organoids containing otic vesicle-like structures with cells resembling cochlear and vestibular cell types, although functional cochlear outer HCs with electromotility or fully functional inner HCs have not yet been conclusively demonstrated.

Despite these advances, the stepwise induction of PSCs to establish inner ear HC organoids in these studies resulted in complex cell compositions, with a significant proportion of mesenchymal cells and only a small percentage of HCs, and required lengthy and cumbersome culture processes [22, 23]. Moreover, these methods fail to induce the differentiation of inner and outer HCs in cochlear organoids and the differentiation of type I and II HCs in vestibular organoids. Subsequent research using different cell sources has continuously refined the methods for generating IEOs, thus further advancing our understanding of inner ear developmental mechanisms and allowing the exploration of therapeutic strategies for hearing disorders (Table 1).

**Table 1:** Cell sources for IEOs

Year	Ref.	Cell type	Highlights	Limitations
2010	[19]	Mouse PSCs	a. Allow for the development of a stepwise induction method	a. Only 1%–2% of cells convert into HCs
			b. Allow the induction of HC-like cells with stereocilia	b. Limited maturation of induced HCs
			c. Assembly into epithelial clusters	c. The use of exogenous tissue limits routine application
2014	[24]	Mouse ESCs	a. Allow the development of a 3D mouse ESC culture system	a. Asynchronous differentiation caused by cell heterogeneity
			b. Reduce the impact of exogenous tissue	b. Only vestibular sensory organ-like structures can be generated
			c. Improved stem cell conversion rate	
2016	[25]	Mouse ESCs	a. Sensory HCs can be induced alongside neuronal populations	a. Only allow the generation of vestibular HCs and not cochlear HCs
			b. The system provides essential bone morphogenetic protein signaling for non-neural ectoderm induction	
2017	[16]	Human PSCs	a. Allow the induction of IEOs	a. Only allow the generation of vestibular sensory structures and not cochlear HCs
			b. The induced HCs exhibit functional electrophysiological properties	
			c. In this system, there is no need for extra bone morphogenetic protein 4 for non-neural ectoderm	
2018	[20]	Human PSCs	a. Optimization of the method of Koehler *et al*. [16]	a. Only outer HCs can be generated, not inner HCs
			b. This system uses a gradual induction technique to generate IEOs	b. The induced IEOs lack the typical intricate structure of the inner ear
			c. The IEOs contain both cochlear and vestibular cell types	c. The vestibular and cochlear HCs are immature
				d. Low K^+^ currents in induced HCs
				e. Unverified normal synaptic functions
2023	[15]	Human PSCs	a. Enhance Sonic Hedgehog/Wingless-Int-1 (Wnt) signaling for ventral gene expression	a. Limited evidence for full inner HC identity
			b. Production of cochlear HCs with morphology similar to *in vivo* HCs	b. Lack of the typical mosaic HC arrangement
			c. Functional properties resembling both outer HCs and inner HCs	c. Absence of central nervous system components
2007	[26]	Mouse TSPs	a. Successful isolation of stem cells from the organ of Corti	a. Limited maturation of induced HC-like cells
			b. Stimulation of Lgr5 up-regulation using growth factors	b. Sphere induction declines with cell age
			c. Formation of sphere-shaped and HC-like cells	
2018	[27]	Human TSPs	a. Expansion of hTSPs from the fetal cochlea	a. 3D cellular arrangement limits microscopy and electrophysiology experiments
			b. First use of Matrigel for TSP-derived IEOs	b. Lack of synaptic connections with neuron-like cells
			c. Allow the formation of both sensory HCs and supporting cells (SCs)	c. Lower yield and expandability compared to PSCs
2023	[17]	Mouse TSPs	a. Co-culture of spiral ganglion neuron explants with TSP-induced IEOs	a. Unable to mimic the *in vivo* polarity in the organ of Corti
			b. Establishment of functional synaptic cochlear organoids	b. Limited modeling of HC-SC arrangement
			c. Modeling of peripheral auditory circuitry	c. Induced HCs mimic development until day 7
				d. Lack of immune system microenvironment and interactions

IEOs, inner ear organoids; Ref, reference; PSCs, pluripotent stem cells; HCs, hair cells; ESCs, embryonic stem cells; TSPs, tissue-specific progenitors; hTSPs, human tissue-specific progenitors

#### TSPs

Considerable progress has been made in generating IEOs using TSPs. Compared with PSCs, TSPs offer a high degree of homology with the epithelium [28], require fewer regulatory steps [29], and provide more controlled differentiation pathways (Table 2). Reports indicate that a subtype of inner ear progenitor cells can re-enter the cell cycle and differentiate into HCs under certain conditions [30]. These progenitors are characterized by the expression of Lgr5 (Leucine-rich repeat-containing G-protein coupled receptor 5), a Wnt-responsive molecule [31]. The Wnt signaling pathway plays a crucial role in various biological processes, including cell differentiation, proliferation, and migration [32]. Inner ear Lgr5+ progenitor cells are abundant in young animals and possess the capacity for self-renewal and differentiation, thus making them a promising source for inner ear HC regeneration [33].

**Table 2:** Comparison of different cell sources for IEOs

Cell Source	Advantages	Limitations	References
ESCs	a. Pluripotency allows differentiation into multiple cell types	a. Ethical concerns	[23, 29, 35–37]
	b. Well-established protocols	b. Complex differentiation protocols requiring multiple steps	
	c. Higher yield of organoids	c. Heterogeneous cellular composition	
	d. Longer lifespan in culture	d. Risk of teratoma formation	
iPSCs	a. Patient-specific modeling is possible	a. Variable reprogramming efficiency	[38–42]
	b. Disease-specific mutations can be studied	b. Reprogramming artifacts	
	c. No ethical concerns	c. Complex differentiation protocols requiring multiple steps	
	d. Can model genetic hearing disorders	d. Costly and time-consuming	
TSPs	a. Higher homology with target epithelium	a. Limited expansion capacity	[22, 28, 43, 44]
	b. More direct differentiation pathway	b. Reduced yield compared to PSCs	
	c. More controlled differentiation	c. Source tissue availability is limited	
	d. Better cellular organization	d. Declines in proliferative capacity with age	
	e. More mature functional properties	e. Challenges in maintaining stemness	

ESCs, embryonic stem cells; iPSCs, induced pluripotent stem cells; TSPs, tissue-specific progenitors

Oshima *et al*. were the first to propose that stimulating Lgr5 expression through growth factors and inducing the expansion of Lgr5+ stem cells isolated from cochlear tissue could lead to the formation of sphere-like structures resembling newly-formed HCs. These cells regain proliferative potential in a 3D culture system and ultimately differentiate into HC-like cells [26]. Roccio *et al*. expanded tissue-specific HC progenitors extracted from the fetal cochlea in a specialized 3D culture system, and these cells exhibited epithelial organization and polarity and differentiated into HC-like cells. This method overcame the influence of genetic background, making it suitable for *in vitro* ototoxic drug screening [27, 34]. However, challenges remain, such as low cell expansion and yield, incomplete maturation of tissue structure and function, and a lack of neuron-HC interactions. Xia *et al*. optimized the proliferation system for Lgr5+ cochlear progenitor cells (CPCs) and found that supplementing the proliferation medium with CHIR99021 and lysophosphatidic acid maximizes progenitor cell sphere formation. In that study, the cochlear HC organoids were also co-cultured with spiral ganglion neurons, thus establishing a neuron-innervated cochlear organoid model [17].

### The Extracellular Matrix (ECM)

#### Traditional Matrix Materials

The construction of IEOs primarily uses Matrigel as the matrix scaffold [17, 20]. Matrigel is a thermosensitive and biologically-derived matrix that exists as a viscous liquid at 4 °C and forms a 3D gel structure at 37 °C. It is composed of a complex mixture of laminin, type IV collagen, entactin, perlecan, and various growth factors [45, 46]. Matrigel is able to mimic the ECM environment *in vivo*, thus providing mechanical support and biochemical cues to promote cell growth, differentiation, and regeneration [47–49]. However, its mechanical properties, such as elasticity and hardness, are uncontrollable, and it carries potential immunogenicity and tumorigenic risks, thus making it unsuitable for 3D printing and certain tissue engineering applications [45, 46]. Nevertheless, Matrigel is widely used in cell culture and biomedical research, particularly in the fields of cell differentiation, proliferation, and regeneration [7, 45, 50]. Research has shown that ECM signals from Matrigel can activate the Ras homolog family member A/Yes-associated protein (YAP)/β-catenin mechanotransduction axis, thus promoting the proliferation of inner ear progenitor cells [51].

#### Emerging Matrix Materials

With advances in the material sciences, hydrogels have increasingly been applied as matrix scaffolds for organoid model construction [52]. Synthetic hydrogels, such as polyethylene glycol and polyacrylic acid, are gaining attention due to their well-defined composition, stable mechanical properties, and ability to be adjusted according to experimental needs [53–55]. Natural hydrogels, on the other hand, are rich in arginine-glycine-aspartic acid (RGD) sequences that support cell migration and adhesion, thus preserving cell viability. After purification and chemical modification, these natural hydrogels can maintain stable and uniform physicochemical properties, meet biocompatibility requirements, and fulfill bioengineering operational needs. By adjusting the degree of cross-linking, different tissue stiffnesses can be simulated, and this has significant potential for both scientific research and clinical applications [53, 56]. Gelatin methacrylate (GelMA) hydrogels, which are derived from gelatin, exhibit superior biocompatibility and can be rapidly cross-linked into a stable gel structure upon exposure to 405 nm wavelength light, thus allowing for precise control over the hydrogel’s physicochemical properties [57]. In addition, hyaluronic acid (HA), a naturally-occurring glycosaminoglycan, can delay hydrogel degradation, thus enhancing matrix stability and water retention [58]. Li *et al*. [59] successfully constructed a composite hydrogel system using GelMA, RGD sequences, and HA, and this facilitated the 3D culture of CPCs. By adjusting the concentration of GelMA within the composite hydrogel, the stiffness of the hydrogel can be finely tuned, and they showed that moderate mechanical stress promotes CPC proliferation through the integrin α3/F-actin cytoskeleton/YAP signaling axis, while higher mechanical stress effectively drives the differentiation of cochlear stem cells into HCs *via* the Ca^2+^/extracellular signal-regulated kinase 1/2/Kruppel-like factor 2 pathway.

**Fig. 2:**
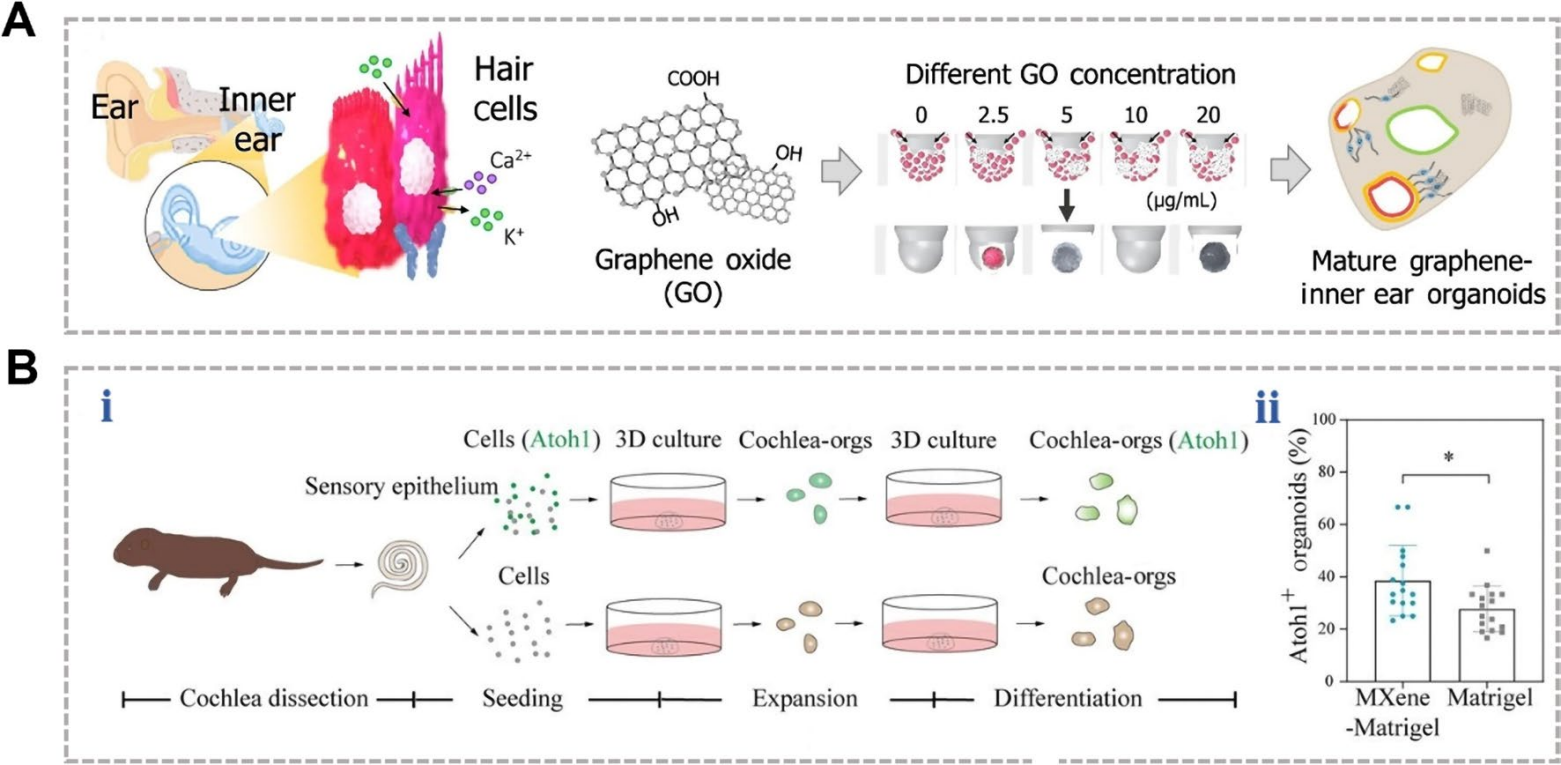
Emerging biomaterials enhance HC development in IEOs through improved substrate properties. **A** Schematic showing the development of graphene-IEOs at varying concentrations of graphene (0, 2.5, 5, 10, and 20 μg/mL). Reproduced with permission [61]. Copyright 2023, American Chemical Society. **B** (i) Summary of the generation process for cochlear HCs *via* differentiation within cochlear organoids. (ii) Comparison of Atoh1-GFP+ organoid formation efficiency between the MXene-Matrigel and Matrigel-only groups (*n* = 16, data represent four independent experiments). Reproduced under terms of the CC‐BY license [62]. Copyright 2022, The Authors, published by Wiley-VCH.

Emerging materials like graphene–which is known for its exceptional electrical conductivity, thermal conductivity, and biocompatibility–are gradually being applied in biomedicine. The surface functional groups and topological structures of these materials guide neural repair and regeneration, making them suitable for drug delivery and bioscaffold manufacturing [60]. Park *et al*. [61] cultured mouse ESCs using graphene oxide and found that graphene oxide enhanced cell-ECM interactions and promoted the formation of gap junctions between cells and thus facilitated the differentiation and maturation of HCs (Fig. 2A). Zhang *et al*. [62] incorporated Ti_3_C_2_T_x_ MXene nanomaterials into Matrigel to modulate the development of cochlear organoids, and this promoted the formation and maturation of HCs in these organoids (Fig. 2B). In addition, light with wavelengths exceeding 800 nm can penetrate the tympanic membrane, and photobiomodulation has been shown to promote the colonization and differentiation of mouse ESCs in sensory epithelia [63]. Hu *et al*. successfully fabricated polypyrrole-polydopamine nanofibers with excellent electrical conductivity and biocompatibility, and they incorporated these nanofibers into Matrigel hydrogel as a dopant modification [64, 65]. This modification endowed the hydrogel with enhanced electrical conductivity, adhesion, and hydrophilicity, thus promoting the maturation and functionalization of IEOs [66–68].

The application of novel hydrogels and conductive materials has significantly improved organoid culture systems, making their composition more precise and controllable. These materials better simulate the mechanical changes that occur during *in vivo* development and provide surface structures and electrical field stimuli within the microenvironment, both of which are essential for the differentiation of sensory HCs and for promoting the normal development and functionalization of these cells.

### Microenvironmental Regulation

#### Biochemical Microenvironment Control

Over the past few decades, research has revealed that various physical, chemical, and biological properties of the extracellular microenvironment play a crucial role in stem cell behavior and regeneration [69–72]. The cultivation and maintenance of IEOs rely heavily on the ability to control the biochemical microenvironment. Different biochemical signals, both endogenous and exogenous, are critical for the formation and homeostasis of organoids [73]. During the organoid culture process, exposing the progenitor cell population to specific morphogens at specific time points activates the required developmental signaling pathways, thus triggering the self-organization process. This process requires the addition of essential components to compensate for the limitations of *in vitro* conditions [74], and these components mainly include growth factors such as epidermal growth factor, fibroblast growth factor, bone morphogenetic protein, B27, cytokines (including Wnt signal activators), hormones, and small molecule inhibitors [4, 75, 76]. Previous studies have suggested that epidermal growth factor, insulin-like growth factor I, and β-fibroblast growth factor are involved in inner ear development due to their mitogenic properties, their ability to promote cell survival, and their ability to induce specific cell phenotypes [77, 78]. McLean *et al*. [18] used organoid models to screen for small-molecule compounds that efficiently induced HC regeneration and found that CHIR99021, a glycogen synthase kinase 3β inhibitor, and valproic acid, which acts as a histone deacetylase inhibitor, effectively promote the expansion of IEOs. They also found that 2-phospho-L-ascorbic acid, a stable derivative of vitamin C, along with the inhibitor of activin receptor-like kinase-5 (the transforming growth factor beta receptor), referred to as 616452, further enhances the proliferation of Lgr5+ supporting cells.

In the cultivation of IEOs, it is essential to precisely regulate the biochemical microenvironment in order to mimic the natural signaling processes occurring *in vivo*. This often requires careful design of the culture medium to ensure that cells receive the appropriate signals at the right time. For example, certain growth factors may need to be added at specific stages of the culture in order to induce desired cell behaviors or tissue formation, thereby optimizing the organoid differentiation and organization processes. In addition, the concentration and duration of these components must be carefully adjusted to accurately replicate the signal transduction processes that occur *in vivo*.

#### Control of the Biophysical Microenvironment

The uniqueness of the auditory system necessitates not only a precise simulation of its biochemical environment, but also an accurate replication of the complex physical environment surrounding the auditory organs during the cultivation of IEOs. Mechanical stimulation plays an indispensable role in the development of IEOs, and mechanical signals mediated through cell-ECM interactions are crucial for the formation of organs such as the brain, lungs, kidneys, muscles, blood vessels, mammary glands, cartilage, and bones [71, 79]. In the cochlear organoid field, researchers have developed a GelMA-HA-RGD hydrogel system with tunable mechanical properties. Using 3D bioprinting technology, they created spiral-shaped cochlear organoids and successfully demonstrated that dynamic mechanical forces from the ECM promote the formation of sensory epithelia, thus laying the groundwork for the role of mechanical properties in the culture of organoids [59]. Future research on IEOs should further integrate microfluidic technology to simulate the lymph flow within the cochlea and thus more accurately recreate the *in vivo* microenvironment of the cochlea. In addition, simulating the ionic polarity differences between the apical and basal chambers of HCs can more precisely reflect the physiological conditions of cochlear sensory epithelia, and co-culturing these organoids with spiral ganglion neurons can enhance the structural fusion and functional integration of auditory units.

**Fig. 3:**
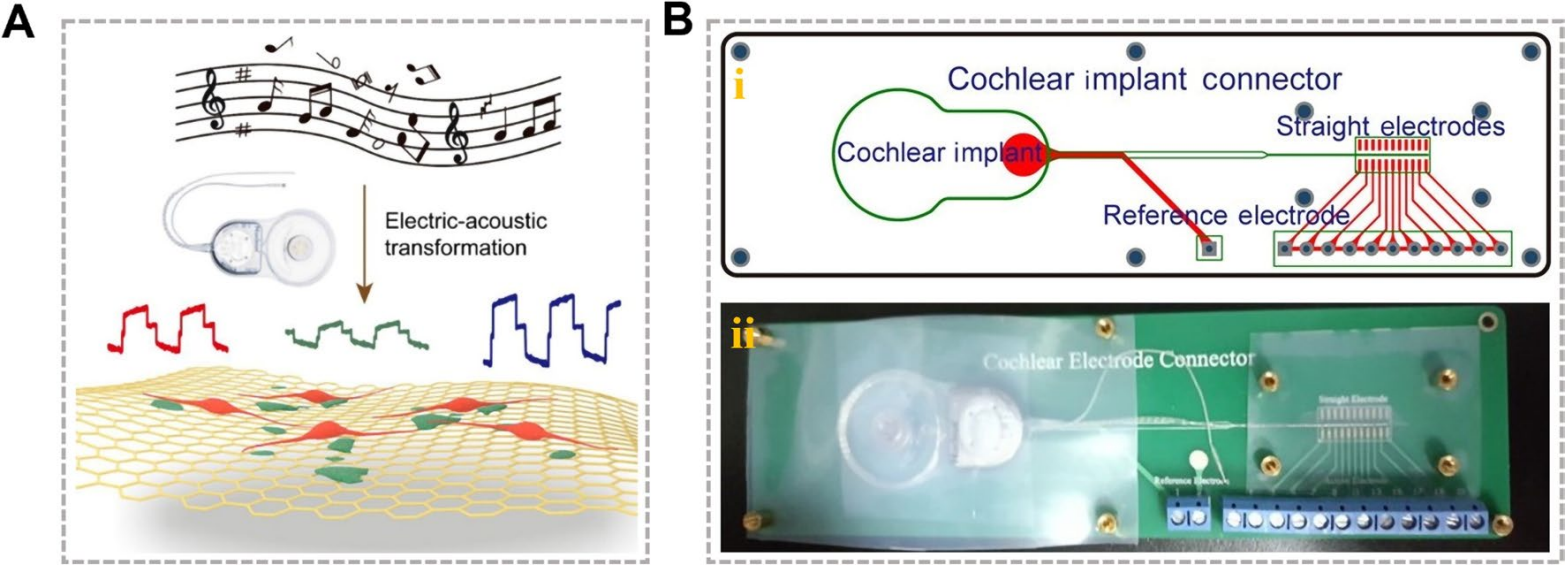
Electric-acoustic stimulation enhances IEO development by mimicking physiological signal transduction. **A** Schematic of electric-acoustic stimulation transformation. Musical notes (upper) represent acoustic signals converted to electrical signals *via* a cochlear implant (middle), which are then transmitted to a simulated neural network on a graphene-based substrate (lower). The colored waveforms below the cochlear implant represent different electric signals corresponding to acoustic frequencies, showing the transformation from acoustic to electrical signals, ultimately interfacing with neural-like structures. **B** (i) Schematic and (ii) photograph of the printed circuit board connected to the cochlear implant. Reproduced with permission [85]. Copyright 2019, American Chemical Society.

In addition to mechanical stimulation, other forms of physical stimuli also play critical roles. Particularly in the construction of IEOs, considering the inherent complexity of the auditory system, where sound waves are converted into electrical signals, physical stimulation becomes especially important [80–82]. The electrical potential gradient around cells has been shown to promote neuronal differentiation, suggesting that electrical stimulation is a potent technique to manipulate excitable cells such as stem cells [83, 84]. Cochlear implants represent another technology based on electrical stimulation, where an electronic device is implanted to stimulate the auditory nerve, thus restoring hearing in patients with hearing impairments. Chai *et al*. have developed an electric-acoustic stimulation system that simulates the combined effects of sound waves and electrical currents in the inner ear under physiological conditions (Fig. 3) [66, 85–87]. The sound wave stimulation activates inner ear sensory cells, causing morphological and functional changes, while the electrical currents significantly enhance cell-to-cell interactions and strengthen intercellular signaling. In addition, this type of stimulation has been shown to affect the gene expression and protein synthesis of IEOs, thus laying the theoretical foundation for using electric-acoustic stimulation to regulate the growth and development of IEOs.

## Applications of IEOs

### Drug Screening

The high failure rate of new drug development in clinical trials is largely due to an incomplete understanding of fundamental human pathophysiology and its underlying mechanisms. Traditional drug screening often relies on animal models, but IEO models can more accurately simulate the physiological environment of the human inner ear. This allows for the direct screening of drugs that may protect inner ear cells, thereby improving the efficiency of drug development. Park *et al*. [61] discovered that HCs from IEOs, generated from mouse ESCs, exhibit similar responses to ototoxic drugs like cisplatin as seen *in vivo*, suggesting that IEO models can reliably reflect the sensitivity of human inner ear cells to drugs. Kurihara *et al*. [88] further showed that neurons in spiral ganglion organoids derived from human iPSCs are significantly damaged by ouabain and cisplatin. In addition, Xia *et al*. [17] demonstrated that the epidermal growth factor, fibroblast growth factor, insulin-like growth factor I, CHIR99021, and lysophosphatidic acid system has a strong regenerative effect on cochlear organoids and cochlear explants. In another study using a Pou4f3^DTR/+^ mouse model, organoid-derived HCs underwent apoptosis following diphtheria toxin treatment, thus providing a robust platform for studying ototoxic drugs and developing protective therapies for the inner ear [89]. These studies indicate that IEOs can faithfully recapitulate the responses of *in vivo* CPCs and HCs to drugs and damaging factors, and they can be used to explore congenital abnormalities in inner ear development, test ototoxicity, and screen for protective and regenerative drugs.

### Disease Modeling for Hearing Loss

The drug discovery and development process is lengthy, and rapidly identifying the right research direction and the most appropriate research methods can significantly shorten this timeline. Thus, selecting a suitable disease model is critical in the early stages of drug discovery. Traditional animal models, while helpful in studying hearing loss, have limitations due to the physiological and genetic differences between humans and animals. IEO models, however, simulate the 3D structure and physiological environment of the human inner ear, offering a more accurate and efficient research platform.

Researchers have used IEO models to simulate the pathological processes of genetic hearing loss *in vitro* and to investigate how gene mutations affect inner ear cell function, thereby uncovering the molecular mechanisms behind hearing loss. For example, Nie *et al*. [90] used human PSC-derived IEOs to study how mutations in *CHD7* (chromodomain helicase DNA binding protein 7) impact inner ear development and found that *CHD7* plays a critical role in regulating human otic lineage specification and HC differentiation. This research provides new insights into the mechanisms of hearing loss and lays the foundation for developing gene therapy approaches. Studies on cochlear progenitor organoids revealed that the regulation of the lin-28 homolog B/lethal-7 microRNA signaling pathway, alongside downstream signaling pathways, significantly promotes cochlear HC regeneration [91–93]. In addition, genes such as hypermethylated in cancer 1, follistatin, *Net1*, and *Gpm6b*, along with novel γ-secretase inhibitors like compound 3 and compound 8, contribute to this regenerative process [94–98].

The application of IEO models has expanded with the integration of high-throughput screening techniques and single-cell sequencing technologies. Ueda *et al*. [99] used single-cell RNA sequencing to analyze the developmental trajectories of prosensory cells in human IEOs. Similarly, Tang *et al*. [100] developed a cochlear organoid model with type II transmembrane serine protease knockout and showed through single-cell RNA sequencing that this knockout leads to the decreased expression of Ca^2+^-binding proteins, reduced numbers of Ca^2+^-activated K^+^ channels, and ultimately HC apoptosis due to ionic imbalance. This research method not only uncovers the specific effects of gene mutations on inner ear cells, but also provides new therapeutic targets. For example, Liu *et al*. [101] applied a cochlear organoid platform combined with high-throughput screening technology to identify the vascular endothelial growth factor receptor - mitogen-activated protein kinase kinase - transforming growth factor beta 1 signaling pathway, which promotes HC reprogramming, and they confirmed that the vascular endothelial growth factor receptor inhibitor regorafenib promotes HC differentiation and maturation *via* the mitogen-activated protein kinase kinase pathway. This methodology has significantly improved the efficiency of drug screening and has led to the discovery of new therapeutic drugs. With the continued development of organoid technology, researchers can now use these models to study diseases more efficiently.

While animal models have contributed significantly to our understanding of inner ear development and pathology, IEOs offer an unparalleled platform for studying the human-specific aspects of inner ear biology. Given the ethical and practical constraints of obtaining staged human fetal tissues, organoids derived from human PSCs represent a unique opportunity to investigate human inner ear development and disease mechanisms in ways previously impossible. This is particularly valuable for understanding conditions where human pathophysiology may differ from animal models due to species-specific developmental trajectories or genetic backgrounds.

### Personalized Medicine

**Fig. 4:**
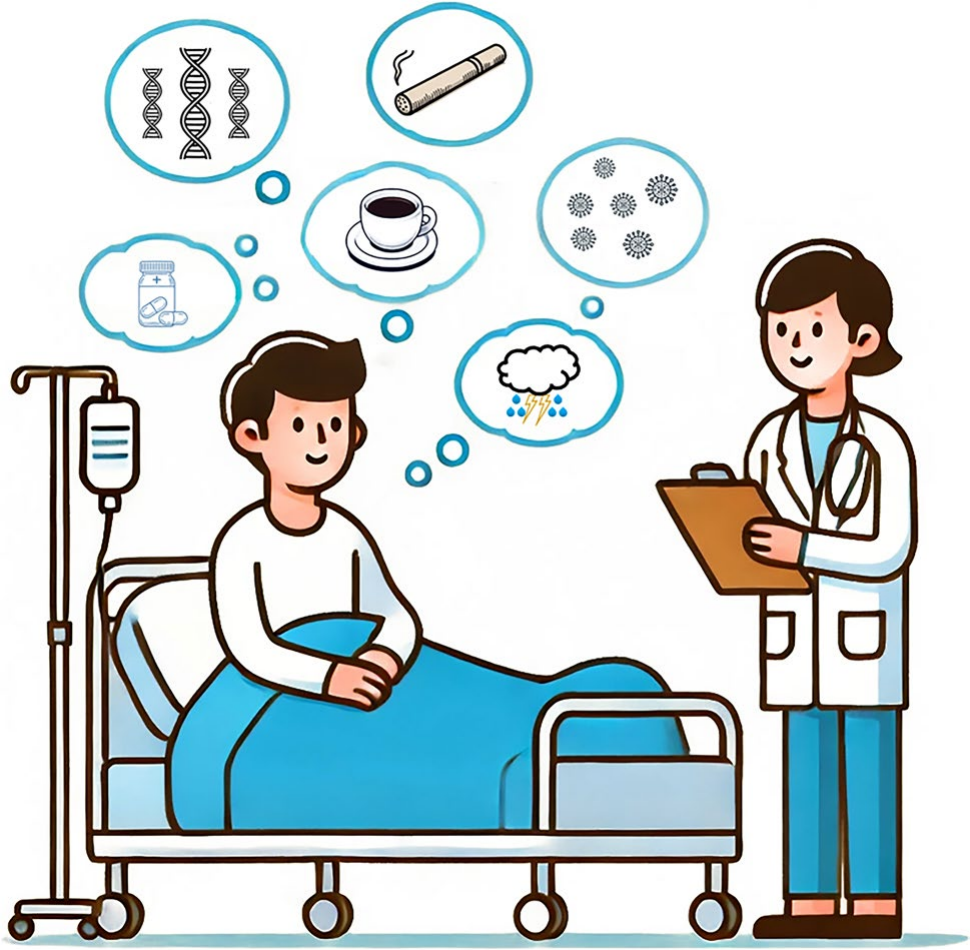
Cartoon of a personalized medicine approach, where the biological information, lifestyle, and environmental factors of a patient are considered. The thought bubbles above the patient represent various health influences. The physician standing at the bedside records these aspects, emphasizing a holistic view in individualized healthcare planning.

Personalized medicine is a customized approach to healthcare based on the biological information, lifestyle, and environmental factors of an individual (Fig. 4). It aims to maximize treatment efficacy, minimize side-effects, and lower costs by offering targeted diagnostics and treatments while also preventing disease and improving the quality of life for patients [102]. This medical model relies on advanced technologies and large-scale data analysis such as DNA sequencing, proteomics, medical imaging, and bioinformatics [103]. Currently, human iPSCs can be derived from the cells of an individual in order to generate corresponding tissues or organoids [104]. In comparison to animal models, these methods show greater advantages, particularly when significant evolutionary or physiological differences exist, and they offer new prospects for precision and personalized medicine [105]. By constructing IEO models from the stem cells of a patient, researchers can investigate the impact of specific genetic backgrounds on inner ear disorders, develop tailored therapeutic strategies, and evaluate individual responses to treatments. This ultimately supports the goals of precision medicine and enables the provision of more effective treatment options.

### Challenges and Future Outlook

In recent years, breakthroughs in 3D culture technologies have accelerated the development of drugs for treating hearing loss, and organoid technology is now seen as a cutting-edge tool for developing *in vitro* models to guide drug discovery. These micro-organoid models, developed from advanced technologies and materials, can simulate genetic models of hearing loss and can be used for high-throughput screening of therapies for sensorineural hearing loss. This offers efficient research tools and models to help restore auditory function, potentially advancing hearing restoration research and clinical applications. Through these innovative methods, researchers can better understand auditory system development and pathological mechanisms, thus driving the development of more effective treatments.

It is important to acknowledge that current IEO studies have yet to provide substantial novel mechanistic insights into inner ear development beyond what has already been established through *in vivo* mouse models. Most signaling pathway modulators applied in organoid protocols, such as Wnt agonists and bone morphogenetic protein inhibitors, were initially identified through *in vivo* genetic studies rather than through organoid systems. This dependency on knowledge derived from animal models raises questions about the unique biological advantages of organoid systems for developmental mechanism discovery compared to traditional model organisms. Moving forward, the field must focus on leveraging the human-specific aspects of organoids to uncover mechanisms that might be distinct from those in animal models, rather than simply replicating findings from *in vivo* studies.

Despite their potential, IEOs face many challenges. As an emerging technology, no method to develop organoids has yet been developed to fully replicate the inner ear *in vitro*. Moreover, the heterogeneity of organoids derived from different sources requires breakthroughs in cost reduction and production efficiency to ensure reproducibility and stability. In the future, integrating microfluidics technology may improve throughput [22], and machine learning might be applied to handle the increasing amounts of data from these studies [106, 107]. Another challenge is the complexity and variability of natural ECM components, which makes controlling the culture environment difficult and reduces experimental reproducibility. Recently introduced synthetic hydrogels with defined chemical components offer better control over the biochemical and mechanical properties of the environment, but their bioactivity needs to be improved. Therefore, regulating the physicochemical properties of the culture matrices is crucial. In addition, current IEOs typically generate vestibular-like structures and immature cochlear HCs, lacking *in vitro* blood vessel system simulation. Inducing and differentiating cell types with cochlear HC characteristics, ensuring that these cells form intercellular connections, and recreating vascular networks within organoids to enhance the precision of physiological simulation are key goals that must be addressed in future therapeutic applications for hearing loss.
